# Enhancing Pain Management and Psychological Recovery in Earthquake Victims: The Role of Continuous Regional Analgesic Techniques Assessed by QoR-15 †

**DOI:** 10.3390/diagnostics14232678

**Published:** 2024-11-27

**Authors:** Ergun Mendes, Ozal Adiyeke, Onur Sarban, Melih Civan, Okyar Altas, Alperen Korucu, Funda Gumus Ozcan, Gozen Oksuz

**Affiliations:** 1 Department of Anesthesiology and Reanimation, Koç University Faculty of Medicine Hospital, Istanbul 34010, Turkey; 2 Department of Anesthesiology and Reanimation, University of Health Sciences, Basaksehir Cam and Sakura City Hospital, Istanbul 34480, Turkey; ozaladiyeke@gmail.com (O.A.); fgumus@hotmail.com (F.G.O.); 3 Department of Anesthesiology and Reanimation, University Hospitals of Sussex Nhs Trust, Brighton BN11 2DH, UK; onursarban@hotmail.com; 4 Department of Orthopedic, University of Health Sciences, Basaksehir Cam and Sakura City Hospital, Istanbul 34480, Turkey; melihcivan@gmail.com; 5 Department of Hand Surgery, University of Health Sciences, Basaksehir Cam and Sakura City Hospital, Istanbul 34480, Turkey; okyaraltas@gmail.com; 6 Department of Orthopedic, Silivri State Hospital, Istanbul 34570, Turkey; dralperenkorucu@gmail.com; 7 Department of Anesthesiology and Reanimation, Faculty of Medicine, Kahramanmaras Sutcu Imam University, Kahramanmaras 46050, Turkey; gozencoskun@gmail.com

**Keywords:** earthquake, compartment syndrome, peripheral nerve catheterization, QoR-15, crush syndrome

## Abstract

Background/Objectives: After the earthquakes in Turkey, many citizens were injured, and a long ongoing process requiring physiological and psychological treatments began. The aim of this study was to observe the pain and psychological changes in earthquake victims in light of the QoR-15 score. Methods: After approval by the local ethics committee (Decision No. 2023-194), earthquake victims with catheters in trauma and reconstructive surgery were retrospectively evaluated. Demographic and catheterization data were collected. Baseline, 24 h, 72 h QoR-15 (Quality of Recovery-15), and VAS (Visual Analog Scala) scores were compared for changes over time. Results: A total of 40 catheters were placed in 29 patients (after the exclusion of children, 36 catheters were evaluated in 26 (15 w/11 m) patients). The mean age of the patients was 35.57 ± 13.69 years, and the duration of catheterization was 8 (4.25–12.75) days. An infusion of 0.1% bupivacaine 0.5–1 mg/kg/24 h was started routinely. The QoR-15 scores of the patients at baseline, 24 h, and 72 h were 80.45 ± 17.76, 95.27 ± 15.16, and 101.06 ± 15.52, respectively (*p* < 0.001). The VAS scores of the patients at baseline, 24 h, and 72 h were 4.61 ± 1.41, 1.79 ± 1.36, and 0.76 ± 0.86, respectively (*p* < 0.001). Conclusions: In this study, a significant improvement in QoR-15 and VAS scores was achieved as a result of catheter insertion in earthquake victims. Considering that post-traumatic injuries require repeated surgeries and that pain aggravates the existing psychological state, it can be said that catheterization is beneficial.

## 1. Introduction

On 6 February 2023, two separate large earthquakes occurred in Turkey, centered on Kahramanmaraş, with a magnitude of 7.8 at 04:17 and a magnitude of 7.5 at 13:24 [1]. After these earthquakes, many buildings collapsed, and many of our citizens were buried under the rubble. After search and rescue efforts, many injured people were pulled from under the rubble [2].

The most common problem in victims trapped under rubble is crush syndrome. Once this pressure is removed, tissue integrity is lost and acute compartment syndrome (ACS) occurs secondary to inflammation and progressive tissue edema. To prevent tissue loss in the acute phase of these patients, a fasciotomy is performed to reduce tissue pressure [3].

Earthquakes, one of the most devastating natural disasters, cause crush injuries under collapsed structures, resulting in a wide range of injuries [3]. Although 11 cities were affected in the acute phase, the life of the whole country was affected by the extended care needs of the patients and the safe evacuation of the area. Our hospital (Başakşehir Çam and Sakura City Hospital, Istanbul, Turkey) acted as the upper center in receiving patients with the help of air transport.

Continuous regional anesthesia techniques, including peripheral nerve blocks, effectively manage pain in earthquake victims, reducing opioid use and improving recovery. Studies highlight their efficacy in disaster settings, as seen during the 2018 Lombok earthquake [2,4,5].

The aim of this study is to observe the physiological and psychological changes in the QoR-15 (Quality of Recovery-15) scoring system in earthquake victims who required surgical intervention and underwent catheterization in our hospital.

## 2. Materials and Methods

### 2.1. Study Design

After local ethics committee approval, patients undergoing trauma and reconstructive surgery were retrospectively evaluated. Patient demographics were determined according to the site of the injury and the type of catheter used.

Catheters were placed in patients with pain and sleep problems, a regular debridement program (2 or more), delayed wound healing, and ongoing tissue loss as part of routine care. Catheters were not placed in patients with known local anesthetic (LA) allergies, catheter site infections, or patients who did not accept the procedure. All patients or their legal representatives were fully informed before consent was obtained.

A wide variety of injuries are seen on admission to our hospital, ranging from simple trauma to crush syndrome and extensive tissue damage. Debridement and vacuum-assisted closure (VAC) are routinely planned for patients undergoing amputation and fasciotomy. Once the wound is self-limiting, defect areas are closed with a primer, flap, or graft. After these procedures, patients who achieved tissue integrity were considered to have completed their clinical treatment and were discharged or transferred to rehabilitation.

Routine anesthetic approach for debridement surgery: after induction, 0.5 mg/kg ketamine and 0.5 mg/kg propofol with unpressurized supraglottic devices were the first choice. At a MAC of 2% sevoflurane, MAC 0.6–1.0 was used in intubated patients with a PEEP of 3–5 cm of water pressure. Patients were routinely given 0.1–0.2 mg of atropine in an anticholinergic dose after induction. In cases such as incompatibility with unpressurized supraglottic devices or suppression of the patient’s spontaneous breathing, rocuronium bromide was added at 0.2–0.3 mg/kg. Pre- and post-operative Doppler images of the patients were assessed for compartment syndrome.

### 2.2. Evaluations

There are 15 questions that assess the patient-reported quality of postoperative recovery using the Turkish version of the QoR-15 [6]; these were collected preoperatively at 24 and 72 h (Baseline, T24, and T72). The QoR-15 scores are divided into two groups. A higher QoR-15 score, with a score between 0 and 10 for each item and a total score of 150, indicates a better quality of recovery. Section A: How did you feel during the last 24 h? Between 0 and 10 (0: never (bad) and 10: always (excellent)) (Q1: Able to breathe easily, Q2: Been able to enjoy food, Q3: Feeling rested, Q4: Have you had a good sleep? Q5: Able to look after personal toilets and hygiene unaided, Q6: Able to communicate with family or friends, Q7: Getting support from hospital doctors and nurses, Q8: Able to return to work or usual home activities, Q9: Feeling comfortable and in control, Q10: Having a feeling of general well-being. Section B: In the last 24 h, have you experienced any of the following? (between 10 and 0, 10: never (excellent) and 0: always (bad)) (Q11: moderate pain, Q12: Severe pain, Q13: Nausea or vomiting; Q14: Feeling worried or anxious; Q15: Feeling sad or depressed. Subheading QoR-15: Physical well-being (pain (Q11–12), physical comfort (Q1–Q2-Q3–Q4–Q13), physical independence (Q5–Q8), and mental well-being (psychological support (Q6–Q7)), emotional state (Q9–Q10–Q14–Q15)). The QoR-15 assessment for these patients at different time points was based on the evaluation conducted at the time of the first catheter placement. No assessments were performed for the catheters placed at later time points.

Patients’ pain was assessed using a Visual Analog Scale (VAS) score between 0 and 10. If the VAS score was greater than 3, paracetamol 10 mg/kg was administered; if the VAS score was greater than 5, tramadol citrate 1 µg/kg was administered. Baseline, 24 h, and 72 h, QoR-15, and VAS scores were compared for changes over time. Patients were evaluated for complications.

### 2.3. Catheterization

All procedures were performed under aseptic conditions using ultrasound guidance with an 8–12 MHz linear probe in the plane. The popliteal block was performed in the lateral decubitus position, the epidural catheter in the sitting position, and all other procedures in the supine position. A space was created by hydrodissection prior to peripheral catheter placement. The catheter was placed using the catheter-through-needle technique.

In the upper extremity, a supraclavicular plexus block catheter (SCPBc) was used for elbow and distal regions, and an interscalene plexus block catheter (ISPBc) for the upper elbow and shoulder regions. The popliteal block catheter (PBc) was used for foot and ankle pain or ischemic pathology, and the suprainguinal fascia iliaca compartment block catheter (SiFICBc) was used for patients with above-knee amputations or large limb tissue defects. The adductor canal block catheter (ACBc) was placed for a sensory block to the saphenous nerve innervation region at and below the knee. Areas with bilateral and/or diffuse tissue defects that were unsuitable for peripheral nerve catheters were converted to epidural catheters (EpiDc).

In brachial plexus catheterization, for SCPBc, the tip of the catheter was placed after imaging the first rib, brachial plexus, and subclavian artery in the supraclavicular region; the marker is placed in the non-fly zone by entering under the inferior cord. For ISPBc, the goal is to place the catheter under the C5 root after sequentially imaging the roots between the anterior and middle scalene muscles when scanning upward from the supraclavicular area.

SiFICBc was placed below the level of the femoral artery and vein at the level of the inguinal ligament; the iliac fascia overlying the nerve was identified, and the probe was then displaced laterally. With the xiphoid facing the sternum, the probe was moved along the medial plane of the inguinal ligament through the anterior superior iliac (SIAS) until the hourglass pattern was identified. The deep circumflex artery is identified at the point where the aponeurotic extensions of the sartorius and the aponeurotic extensions of the internal abdominal muscle are identified. The procedure is performed after identifying the fascia running just below this artery and above the psoas muscle. A catheter is placed under the iliac fascia between the needle insertion point and the psoas muscle.

The PBc is placed midway between the two nerves, just below the junction, after visualizing the posterior tibial and common peroneal nerves between the popliteal artery and the skin just above the knee. The ACBc is placed under the sartorius muscle above the knee, just before the femoral artery and saphenous nerve deepen, and the catheter is placed in the adductor canal. The EpiDc is placed in the L3–L4 or L4–L5 intervertebral space with the patient in the sitting position.

For SCPBc, ISPBc, PBc, and ACBc, 0.1% bupivacaine 0.5 mg/kg/24 h infusion, 1 mL bolus, and 30 min lock-out were used, whereas, for SiFICBc, 0.1% bupivacaine 1 mg/kg/24 h infusion, 2 mL bolus, and 30 min lock-out were routinely used. For EpiDc patients, 0.1% bupivacaine + 4 mcg fentanyl 0.5 mg/kg/24 h, 1 mL bolus, and 30 min lock-out were used. This was later adjusted according to the patients’ needs. Catheters were routinely followed by the pain team after 4 h with the insertion of a patient-controlled analgesia (PCA) device. The infusion rate was determined by dividing the total daily local anesthetic dose by weight. Table 1 shows the catheters used, their location, shape, and infusion rate.

### 2.4. Statistics

The primary outcome of this study is to evaluate whether catheter use improves patient comfort and quality of life in earthquake victims using the QoR-15 composite score. A secondary outcome is to evaluate which parameters within the QoR-15 sub-items show differences.

The SPSS 22.0 package program was used for all analyses. A *p*-value of <0.05 was regarded as significant. The confidence interval was set at 95%. Descriptive statistics are presented as mean ± SD or IQR (25–75%). Categorical variables are presented as the number of cases (*n*) within the time comparison of the QoR-15 score and VAS. The Shapiro–Wilk test was used to test whether quantitative data conformed to a normal distribution. Student’s *t*-tests were used to compare variables that conformed to normal distributions in both groups, while Mann–Whitney U tests were used for non-normal distributions. A one-way repeated measures ANOVA, or Friedman’s test, was used to examine differences between timelines. Friedman’s test and Bonferroni correction were used. Correlations between categorical variables were tested using chi-squared tests.

## 3. Results

### 3.1. Patient Data

A total of 40 catheters were identified in 29 (18 w/11 m) patients (36 catheters in 26 (15 w/11 m) patients after excluding 3 children). The mean age of the patients was 32.72 ± 15.50 and 35.57 ± 13.69 years, and the duration of catheterization was 8 (4.00–12.75) and 8 (4.25–12.75) days (before and after exclusion of children, respectively). After the exclusion of children, the patients had a height of 156.30 ± 42.17, a weight of 73.80 ± 13.92, and a Body Mass Index (BMI) of 25.95 ± 3.87. Post-catheter bupivacaine requirements were 1.01 ± 0.51 mg/day (*n* = 40) and 1.05 ± 0.52 mg/day (*n* = 36) when children were excluded. The type of infusion rate and number of catheters are shown in Table 2 with demographic data.

While two different peripheral catheters were placed simultaneously in three patients, six patients required repeat catheterization in the later stages of treatment. Regarding the types of catheters placed, 16 SiFICBc, 8 SCPBc, 2 ISPBc, 5 PBc, 1 ACBc, and 2 EPc were placed. Multiple catheterizations were performed on three patients: PBc + SiFICBc, SCPBc + SiFICBc, and bilateral SiFICBc. In the postoperative period, nausea and vomiting developed in one patient, catheter dislodgement in six patients, and local skin infection in four patients, while no complications were observed in the other patients.

### 3.2. QoR-15

According to QoR-15, Q5, Q8, Q14, and Q15 were statistically similar at all time points, respectively. (*p* = 0.061, *p* = 0.424, *p* = 0.435, and *p* = 0.695, respectively).

Q1, Q2, Q6, and Q13 were statistically significant at all time points, respectively (*p* = 0.006, *p* = 0.001, and *p* = 0.003). When Q1, Q2, Q6, and Q13 were compared with each other according to their changes over time, the *p* values of Baseline-T24 and Baseline-T72 were found to be statistically significant (*p* = 0.011–*p* = 0.021, *p* = 0.021–*p* = 0.003, *p* = 0.013–*p* = 0.001, and *p* = 0.012–*p* = 0.010, respectively); the *p* values of T24–T72 were found to be statistically similar (*p* = 0.584, *p* = 0.090, *p* = 0.180, and *p* = 0.412, respectively).

Q3, Q4, Q7, and Q11 were statistically highly significant at all time points, respectively (*p* < 0.001, *p* < 0.001), *p* < 0.001, *p* < 0.001, *p* < 0.001). When Q3, Q4, and Q11 were compared with each other according to their changes over time, the *p* values of Baseline-T24 and Baseline-T72 were found to be highly statistically significant (*p* < 0.001); the *p* values of T24–T72 were found to be statistically significant (*p* = 0.005, *p* = 0.028 and *p* = 0.001, respectively). When Q7 was compared according to their changes over time, the *p* values of Baseline-T24 and T24–T72 were found to be statistically significant (*p* = 0.035 and *p* = 0.020; respectively); the *p* value of Baseline-T72 was found to be highly statistically significant (*p* < 0.001).

Q9, Q10, Q12, and Q-Total were highly statistically significant at all time points, respectively (*p* < 0.001, *p* < 0.001, *p* = 0.000), *p* < 0.001, *p* < 0.001). When Q9, Q10, Q12, and Q-Total were compared with each other according to their changes over time, the *p* values of Baseline-T24, T24–T72, and Baseline-T72 were found to be highly statistically significant (*p* < 0.001). Table 3 shows the QoR-15 changes in the patients over time.

### 3.3. VAS Scores

According to VAS scores, they were highly statistically significant (*p* < 0.001). VAS scores were 4.61 ± 1.41, 1.79 ± 1.36, and 0.76 ± 0.86 (Baseline, T24, and T72, respectively). When VAS scores were compared according to their changes over time, the *p* values of Baseline-T24, T24–T72, and Baseline-T72 were found to be highly statistically significant (*p* < 0.001).

**Table 3:** QoR-15 (Quality of Recovery-15) Patient Data.

	Baseline	T24	T72	*p*
Section A				
How did you feel during the last 24 h?				
Between 0 and 10 (0: never (bad) and 10: always (excellent)				
0 1 2 3 4 5 6 7 8 9 10				
Never ------------------------------------------------------------------------------------------------------------------------------------- Always				
0 1 2 3 4 5 6 7 8 9 10				
Q1: Able to breathe easily	8.52 ± 1.85	8.97 ± 1.26	9.06 ± 1.49	0.006 ^a,c^
Q2: Been able to enjoy food	7.03 ± 1.59	7.52 ± 1.37	7.73 ± 1.23	0.006 ^a,c^
Q3: Feeling rested	4.94 ± 1.54	6.79 ± 1.24	7.52 ± 1.62	0.000 ^a,b,c^
Q4: Have had a good sleep	5.06 ± 2.19	7.48 ± 1.50	7.91 ± 1.60	0.000 ^a,b,c^
Q5: Able to look after personal toilet and hygiene unaided	1.55 ± 2.64	1.76 ± 2.81	1.81 ± 1.95	0.061
Q6: Able to communicate with family or friends	8.79 ± 1.57	9.09 ± 1.18	9.18 ± 1.13	0.001 ^a,c^
Q7: Getting support from hospital doctors and nurses	8.09 ± 1.89	8.48 ± 1.48	8.94 ± 1.17	0.000 ^a,b,c^
Q8: Able to return to work or usual home activities	1.42 ± 2.27	1.52 ± 2.19	1.61 ± 2.39	0.424
Q9: Feeling comfortable and in control	4.42 ± 1.93	5.61 ± 1.93	6.33 ± 1.91	0.000 ^a,b,c^
Q10: Having a feeling of general well-being	4.55 ± 1.85	5.67 ± 1.49	6.55 ± 1.80	0.000 ^a,b,c^
Section B				
In the last 24 h, have you experienced any of the following?				
Between 10 and 0, 10: never (excellent) and 0: always (bad)				
10 9 8 7 6 5 4 3 2 1 0				
Never ------------------------------------------------------------------------------------------------------------------------------------Always				
10 9 8 7 6 5 4 3 2 1 0				
Q11: Moderate pain	4.64 ± 1.71	7.00 ± 1.58	7.00 ± 1.33	0.000 ^a,b,c^
Q12: Severe pain	5.06 ± 2.60	7.73 ± 2.48	8.61 ± 1.22	0.000 ^a,b,c^
Q13: Nausea or vomiting	7.33 ± 2.66	8.42 ± 1.54	8.55 ± 1.69	0.003 ^a,c^
Q14: Feeling worried or anxious	4.21 ± 1.78	4.36 ± 1.36	4.76 ± 1.69	0.435 ^b^
Q15: Feeling sad or depressed	4.70 ± 1.44	4.88 ± 1.51	4.79 ± 1.76	0.695
Total	80.45 ± 17.76	95.27 ± 15.16	101.06 ± 15.52	0.000 ^a,b,c^

(^a^) Baseline-T24 (^b^) T24–T72 (^c^) Baseline-T72 (*p* < 0.05 was considered statistically significant) (*p* = 0.000 = *p* < 0.001). Q14: T24–T72 *p* < 0.05, Q1–Q2–Q6–Q13: Baseline-T24 and Baseline-T72 *p* < 0.05, Q3–Q4–Q11: T0–T24 and T0–T72 *p* < 0.001; T24–T72 *p* < 0.05. Q7: T0–T72 *p* < 0.001; T0–T1 and T24–T72 *p* < 0.05, Q9–Q10–Q12–(Q-Total): T0–T24, T0–T72, and T24–T72 *p* < 0.001.

## 4. Discussion

This study has shown us, using the QoR-15 recovery score, that catheter use improves patient comfort and quality of life in earthquake victims. It is the first study in the literature to evaluate catheter use in the same group of post-earthquake trauma patients using the QoR-15. Using these scores, we aimed to assess the impact of catheter use on physical and mental parameters in these patients with a similar trauma history. Earthquake victims, in addition to their physical limitations due to trauma, also experience psychological stress caused by being buried under rubble [7]. Our study is also important because it is the only study that specifically recruited earthquake patients.

Regional anesthesia (RA) techniques can be safely used in disaster situations with challenging environmental conditions and limited resources [8]. RA should be preferred and recommended to patients for post-disaster surgery due to reduced pain and complication rates [9]. Lehavi et al. found that the use of peripheral blocks after the 2015 Nepal earthquake controlled patients’ pain levels, contributed to patients’ quality of recovery, and reduced intensive care unit (ICU) use and oxygen support in the postoperative period. This article describes the author’s patient experience during the first 2 weeks and is not an original article but an editorial note [10]. For earthquake victims, crush injury is a prolonged injury that requires periodic debridement after fasciotomy [8].

The acute injuries of earthquake victims in the acute phase require interventions such as fasciotomies, amputations, and extensive tissue damage in orthopedic and plastic surgery [2]. These patients undergo a chronic process with recurrent interventions requiring repeated debridement. Impaired tissue perfusion, accompanied by constant plasma leakage from this region, leads to a catabolic process with hypovolemia in patients [11]. Despite routine analgesic protocols, these patients have difficulty achieving adequate pain control, primarily due to high analgesic requirements and impaired drug metabolism and excretion due to impaired circulation [4].

Pain control is achieved by reducing the level of afferent input and, consequently, local and systemic mediators and inflammatory stressors [12]. In trauma patients, “rebound pain” may be an additional problem as the pathology persists after peripheral nerve blocks [13]. It has been reported that the incidence of rebound pain after outpatient surgery can reach up to 40% of patients after peripheral nerve block (PNB) resolution [14]. When using single-shot PNB, the use of a higher concentration of LA compared to infusion may break the vasoconstriction developed in the region, causing bolus blood flow and additional tissue loss [15]. Continuous peripheral nerve block (CPNB) is expected to reduce the risk of acute circulatory overload or rebound pain in this area in patients who have been in hospital for a long time and require repeat surgery [16].

QoR-15, the abbreviated form of QoR-40, has been found to be valid, comprehensive, and sufficient [6,17]. We used the QoR-15 form in the study. Overall, the recovery scores for physical well-being (pain and physical comfort) and mental well-being (psychological support) improved. However, there was no improvement in physical independence. While there was an improvement in mental well-being, indicating emotional state, there was no change in (Q14–Q15).

According to our study, there are significant reductions in pain scores in all time periods. It allows the use of long-acting LA in low concentrations, such as 0.1–0.2 mg/mL, depending on the use of a catheter [18]. We see its successful use in a variety of areas, including the use of different doses and LA, different infusions, and bolus levels, suitable for many types of peripheral blocks [12]. CPNB applications in upper extremity burns have resulted in lower patient pain scores and reduced analgesic consumption [19]. Its use in lower extremity amputation surgery has been reported to reduce pain and opioid consumption, as well as reduce the risk of pulmonary complications in patients [20].

There is a general reluctance among surgical teams to use regional techniques because of the perceived risk of ACS following a peripheral block. Although there is no clear evidence that ACS develops due to peripheral block, it has been stated that a pain state that breaks the peripheral block is a red flag. It has been noted that this risk is reduced by a low-concentration infusion of CPNB [21]. These practices remain limited due to the general disruption of health services in disaster situations and the need for constant and close monitoring of catheterized patients, which requires additional effort [22,23]. We assessed patients by Doppler while monitoring our own catheters, and no change in Doppler flow was observed during our regular infusions.

Large tissue defects and amputations are most common in earthquake victims due to lower extremity injuries [5]. The femoral nerve is important for pain control in these patients, most of whom have knee and above-knee injuries [24]. Catheters placed through the femoral region are at risk of infection and dislocation [25]. By using more SiFICB catheters, we have tended to increase the coverage area in these patients, with the lateral femoral cutaneous nerve (LFCN) and possibly the obturator nerve contributing in addition to the femoral nerve [26]. The move to the abdomen as a catheter site is intended to reduce the risk of dislodgement due to infection and patient movement and to increase patient comfort [27]. Even with bilateral SiFICB catheters, pain control was achieved without complications at non-toxic infusion levels due to the low LA concentration (maximum 2 mg/kg/day bupivacaine infusion). In hypovolemic patients with war injuries, blood LA concentrations were found to be low even with high concentrations and volumes of LA in CPNB catheter applications [28,29].

Post-traumatic stress disorder (PTSD) is a condition in which symptoms develop following an extreme traumatic stressor that exceeds the individual’s ability to cope. The psychological impact of the earthquake is significant; the incidence of PTSD in survivors of severe earthquake trauma is as high as 87% [7]. Persistent symptoms lead to re-experiencing the event, increased hypervigilance, and anxiety. The relationship between PTSD and chronic pain has a number of common factors that cause them to coexist (anxiety increases the perception of pain). Persistent reminders of the traumatic event lead to an arousal response, which leads to an avoidance factor. Avoidance of painful activities leads to a loss of physical fitness and increased disability [30].

Neuropathic pain is an important consequence of a crush injury. The literature investigating the relationship between crush injuries and chronic pain is limited [31]. It has been shown that there is a high rate of conversion to chronic pain after acute trauma and that the most important parameter in the development of chronic pain is related to the intensity of the acute pain [32]. Although peripheral block applications have found their place as a guideline for musculoskeletal injuries [33], they are not included in the treatment of neuropathic pain [34]. There is limited research in the form of case reports or case series on the use of catheters. In one recently published study, the use of a catheter in patients with chronic post-amputation pain resulted in more than a twofold improvement [35].

In the post-traumatic period, patients’ psychological processes continue with their ongoing surgical processes. Continuity of pain control is necessary during this period. For these patients, the continuity in the absence of parameters such as pain that reminds them of a possible trauma, seems to be the most important parameter [36]. Thanks to the CPNB application, we minimized the possibility of pain and observed additional improvements in the patients thanks to this control. Our study sheds light on the improvements in patients’ mental and physical parameters thanks to pain control. Although we reached this conclusion specifically in earthquake victims, we believe that the use of CPNB should be considered in trauma patients with ongoing organ damage in similar situations.

Lai et al. noted that evidence of infection following the use of peripheral catheters is rare, reporting a 1% infection rate in their clinical experience, consistent with findings in the literature [37]. Buckenmaier et al. reported a catheter-specific complication rate of 3.7% in 187 military trauma patients, with an average catheter duration of 8 days (ranging from 1 to 33 days). These complications included bending, dislodgement, and superficial infection [38]. In our study, no additional complications occurred except for localized skin infections.

It should be noted that the improvement in patient scores in this study cannot be solely attributed to catheter placement. Various factors, including the overall quality of patient care, postoperative pain management, and psychological support, may contribute to the improvement in scores, in addition to catheter use. Especially in post-traumatic settings, adequate pain control, minimizing infection risk, and providing psychological support play a crucial role in the overall recovery process [5]. In our study, the effects of these factors were considered, and it is believed that the improvement in QoR-15 scores could be attributed to a multidimensional treatment approach.

## 5. Conclusions

In this study, a significant improvement in QoR-15 and VAS scores was achieved as a result of catheter insertion. Considering that post-traumatic injuries require repeated surgery and that pain exacerbates the existing psychological state, catheterization can be considered beneficial.

Limitations: As prospective planning for natural disasters such as earthquakes is not possible, we retrospectively evaluated the routine prospective treatment algorithm. The lower average age in our dataset may be attributed to the vulnerability of elderly individuals in such disasters due to factors like physical frailty, chronic illnesses, and limited mobility.

Additional Note: I offer my patience and condolences to all our citizens affected by the earthquake. I hope that such destructive scenes will be far away from all world communities.

## Figures and Tables

**Table 1:** Type and number of catheters Demographics.

	*n*	Before Exclusion	*n*	After Exclusion
Age	29	32.72 ± 15.50	26	35.57 ± 13.69
Gender (female/male)	29	18/11	26	15/11
Height (cm)	29	150.34 ± 43.69	26	156.30 ± 42.17
Weight (kg)	29	68.89 ± 19,78	26	73.80 ± 13,92
BMI (kg/m^2^)	29	26.05 ± 3.79	26	25.95 ± 3.87
ASA(I/II)	29	24/5	26	21/5
Duration of Catheters IQR (25–75%)	40	8 (4.00–12.75)	36	8 (4.25–12.75)
Catheter side (Left/Right/Epidural)	40	24/14/2	36	22/12/2
Complication type (NV/D/inf/none)	40	1/6/4/29	36	1/6/4/25
Catheter type (Catheter of the patients)				
SiFICBc	16	Case 7–10–11–13–14–16–18(2x)–22–23–25–26–27–28–29	15	Excluded: Case 9
SCPBc	8	Case 2–4–12–15–19–24	6	Excluded: Case 6–21
ISPBc	2	Case 1–4	2	-
PBc	5	Case 3–5–7–8	4	Excluded: Case 9
ACBc	1	Case 5	1	-
EpiDc	2	Case 13–14	2	-
PBc + SiFICBc	2	Case 20	2	-
SCPBc + SiFICBc	2	Case 17	2	-
Bilateral SiFICBc	2	Case 14	2	-
Infusion rate (mg/kg/day)				
ISPBc	2	0.65 ± 0.17	2	0.65 ± 0.17
SCPBc	10	0.58 ± 0.12	8	0.59 ± 0.13
PBc	6	0.67 ± 0.14	5	0.71 ± 0.13
ACBc	1	0.80 ± 0.00	1	0.80 ± 0.00
SiFICBc	19	1.44 ± 0.41	18	1.47 ± 0.41
EpiDc	2	0.50 ± 0.00	2	0.50 ± 0.00
Total	40	1.01 ± 0.51	36	1.05 ± 0.52

(*p* < 0.05 was considered statistically significant) (*p* = 0.000 = *p* < 0.001). BMI: Body mass index, ASA: American Society of Anesthesiologists, NV/D/inf/none: Nausea/Vomiting/Dislocation/infection/none. ISPBc: interscalene brachial plexus catheterization, SCPBc: supraclavicular brachial plexus catheterization, PBc: popliteal block catheterization, ACBc: adductor channel block catheterization, SiFICBc: suprainguinal fascia iliaca compartment block catheterization, EpiDc: epidural catheterization.

**Table 2:** Use of continuous peripheral nerve block (CPNB).

	Position	Coverage	Procedure	Infusion
Supraclavicular Brachial Plexus Catheterization (SCPBc)	Supine In-plane Linear probe (8–12 mHz)	Elbow Below elbow	View of first rib, brachial plexus, and subclavian artery No-fly zone below the lower cordon	0.1% bupivacaine 0.5 mg/kg/24 h 1 mL bolus 30 min lock-out
Interscalene Brachial Plexus Catheterization (ISPBc)	Supine In-plane Linear probe (8–12 mHz)	Above elbow Shoulder joint	In the anterior and middle scalene muscles between the upper and middle brachial trunk	0.1% bupivacaine 0.5 mg/kg/24 h 1 mL bolus 30 min lock-out
Popliteal Block Catheterization (PBc)	Lateral dekubitis (L/R) In-plane Linear probe (8–12 mHz)	Foot Ankle Below knee	Above the popliteal artery between the posterior tibial and common peroneal nerves	0.1% bupivacaine 0.5 mg/kg/24 h 1 mL bolus 30 min lock-out
Adductor Channel Block Catheterization (ACBc)	Supine In-plane Linear probe (8–12 mHz)	Below knee Knee	Below the sartorius muscle Lateral to the femoral artery At the entrance to the adductor canal	0.1% bupivacaine 0.5 mg/kg/24 h 1 mL bolus 30 min lock-out
Suprainguinal Fascia Iliaca Compartment Block Catheterization (SiFICBc)	Supine In-plane Linear probe (8–12 mHz)	Knee Above knee Wide tissue defects	Below the deep circumflex artery The internal abdominal aponeurosis with the sartorius aponeurosis Between the iliacus muscle and the iliac fascia	0.1% bupivacaine 1 mg/kg/24 h 2 mL bolus 30 min lock-out
Epidural Catheterization (EpiDc)	Sitting Tuffier’s line With landmark	Pelvic Lower abdomen Lower extremity	L3–L4 L4–L5	4 mcg Fentanyl + 0.1% bupivacaine (mL) 0.5 mg/kg/24 h (bupivacaine) 1 mL bolus 30 min lock-out

## Data Availability

The data underlying this article will be shared on reasonable request to the corresponding author.
